# Controlled arterial hypotension during resection of cerebral arteriovenous malformations

**DOI:** 10.1186/s12883-021-02362-x

**Published:** 2021-09-06

**Authors:** Katharina Riedel, Marcus Thudium, Azize Boström, Johannes Schramm, Martin Soehle

**Affiliations:** 1 Department of Anaesthesiology and Intensive Care Medicine, University Hospital Bonn, Bonn, Germany; 2 Medical Faculty, University Hospital Bonn, Bonn, Germany; 3 MEDICLIN Robert Janker Hospital, Bonn, Germany

**Keywords:** Blood loss, surgical, Controlled hypotension, Intracranial Arteriovenous malformations, Neurosurgery, Urapidil

## Abstract

**Background:**

Resection of cerebral arteriovenous malformations (AVM) is technically demanding because of size, eloquent location or diffuse nidus. Controlled arterial hypotension (CAH) could facilitate haemostasis. We performed a study to characterize the duration and degree of CAH and to investigate its association with blood loss and outcome.

**Methods:**

We retrospectively analysed intraoperative arterial blood pressure of 56 patients that underwent AVM-resection performed by the same neurosurgeon between 2003 and 2012. Degree of CAH, AVM size, grading and neurological outcome were studied. Patients were divided into two groups, depending on whether CAH was performed (hypotension group) or not (control group).

**Results:**

The hypotension group consisted of 28 patients, which presented with riskier to treat AVMs and a higher Spetzler-Martin grading. CAH was achieved by application of urapidil, increasing anaesthetic depth or a combination thereof. Systolic and mean arterial blood pressure were lowered to 82 ± 7 and 57 ± 7 mmHg, respectively, for a median duration of 58 min [25% percentile: 26 min.; 75% percentile: 107 min]. In the hypotension group, duration of surgery (4.4 ± 1.3 h) was significantly (*p* <  0.001) longer, and median blood loss (500 ml) was significantly (*p* = 0.002) higher than in the control group (3.3 ± 0.9 h and 200 ml, respectively). No case fatalities occurred. CAH was associated with a higher amount of postoperative neurological deficits.

**Conclusions:**

Whether CAH caused neurological deficits or prevented worse outcomes could be clarified by a prospective randomised study, which is regarded as ethically problematic in the context of bleeding. CAH should only be used after strict indication and should be applied as mild and short as possible.

## Background

Blood flow through a vessel is determined by both perfusion pressure and vessel diameter. Whenever a vessel is injured by surgery, it will be occluded by coagulation, ligation or clipping. However, some vessels cannot be occluded immediately which could result in profound blood loss. In these instances, a lowering of perfusion pressure will reduce blood loss and allows the surgeon a better view (and control) of the bleeding source. As a consequence, controlled arterial hypotension (CAH), defined as a lowering of arterial blood pressure (ABP) by pharmacologic measures, is an established procedure [15] in ophthalmic, ear, endoscopic or vascular surgery.

For cerebral vessels, the pathophysiology is at least partially different, since cerebral autoregulation will maintain cerebral blood flow (CBF) despite lowering of arterial blood pressure. Only if ABP drops below the lower threshold of cerebral autoregulation, then CBF will decrease and blood loss through an injured cerebral vessel will be reduced. Anaesthesia affects cerebral autoregulation depending on the hypnotic drugs used: It is maintained during propofol anaesthesia but impaired when using volatile anaesthetics in a dose depending fashion: While cerebral autoregulation is preserved during concentrations up to 1 MAC, it is impaired at higher concentrations (> 1.5 MAC) [32].

Cerebral arteriovenous malformations (AVM) are vascular abnormalities characterized by a direct connection via pathological vessels between arteries and veins without a normal interposed capillary bed. Therefore, vessel resistance is reduced resulting in a low perfusion pressure but high blood flow through the AVM. The autoregulatory curve of the adjacent brain tissue is shifted to the left [15, 22] to compensate for the reduction in perfusion pressure and to prevent cerebral ischemia.

The different anatomical structure of AVMs makes them fragile and difficult or impossible to occlude by coagulation. In addition, cerebral autoregulation is absent in these vessels, which makes controlled arterial hypotension an effective measure to reduce blood flow and blood loss during AVM resection [14, 15, 17, 24]. While CAH might be helpful to control blood loss during surgery, it may cause harm especially when ABP drops below the lower limit of cerebral autoregulation leading to cerebral ischemia and neuronal damage [24].

There is a gap of knowledge about what degree of CAH can be considered safe. Therefore, we analysed the extent of the reduction in blood pressure, as primary objective. Secondary objectives were the duration of CAH, the methods used to induce and terminate CAH, blood loss and neurological outcome. We hypothesized, that the application of CAH does not impair the neurological outcome.

## Methods

All patients who ever harboured an AVM that was removed by the senior neurosurgeon (J.S.) at our institution between 1990 and 2012 were included. Patients for whom no anaesthetic protocol was available were excluded. Based on the anaesthesia records, the intraoperative course of ABP, blood loss, duration of surgery and the methods used to induce, maintain and terminate CAH were studied. Additional information with respect to clinical presentation on admission, AVM characteristics according to the Spetzler-Martin Grading [35], and outcome specified by the modified Rankin Scale (mRS) [28] were retrieved from the local AVM database.

Either a balanced or a total intravenous anaesthesia was performed. All patients received cis-atracurium and remifentanil for muscle relaxation and antinociception, respectively. Patients were intubated and mechanically ventilated. Arterial blood pressure was determined invasively by a radial arterial line, which was referenced to the level of the right cardiac atrium. Any substantial blood loss was replaced by packed red blood cells, and haemoglobin concentration was determined via arterial blood gas analysis whenever it was deemed necessary. The details of the surgical technique are described elsewhere [30]. The neurosurgeon expressed the wish for CAH, usually because of expected difficulties, sometimes because of unexpected severe bleeding. Criteria used by the surgeon to wish for CAH were large size of nidus, high flow AVMs as deducted from the size of feeders, and diffuse periphery of the nidus, i.e. not well-demarcated borders to the healthy brain tissue, or a combination of these three factors. The neuroanaesthetist then discussed with the surgeon whether pre-existing conditions or special circumstances spoke against CAH. Finally, the anaesthetist and the neurosurgeon made a team decision and determined the degree of CAH, i.e. the target ABP as well as the start and end time of CAH. Usually, this decision for CAH was made before the start of surgery and discussed at the beginning of the operation. Depending on the team’s decision, patients were assigned to the hypotension or control group, respectively. The choice of the agents used to induce, maintain and terminate CAH was at the discretion of the anaesthesiologist. By definition, CAH started as soon as a drop in ABP was visible in the anaesthesia record and ended, when ABP rose above the lowered level. The target ABP to be achieved during the CAH-period was documented in the anaesthesia record. If the blood pressure was lowered but the target value - for whatever reason - was not reached, the patient was still left in the hypotension group.

The patient records were searched for evidence of acute renal failure or acute myocardial infarction in the postoperative course. The former was defined as the occurrence of oliguria or the need to perform a renal replacement therapy. The latter was assumed if a cardiologist made the corresponding diagnosis based on clinical symptoms as well as ECG and troponin changes. Neurological complications were defined as occurrence of any non-pre-existing intracerebral haemorrhage or neurological deficit in terms of motor, sensory, visual, or speech deficit. Neurological outcome was assessed immediately postoperative until discharge, as well as 6 months thereafter using the modified Rankin Scale [3].

Statistical analysis was performed using SigmaPlot (version 14.0, Systat Software GmbH, Erkrath, Germany). Data are shown as mean ± standard deviation in case of normal distribution or as median [25, 75% percentile] otherwise. Groups were compared using the unpaired t-test or the Mann-Whitney Rank Sum Test, respectively, and statistical significance was assumed at a *p* <  0.05. To identify predictors of outcome, a Receiver Operating Characteristic (ROC) as well as a Stepwise Regression Analysis were performed.

## Results

### Preoperative data of the study cohort

Of the 226 patients operated on by the senior neurosurgeon JS, 67 operations took place during the period (from 2003) when anaesthesia records were available. In 11 patients in the AVM database, no corresponding anaesthesia protocol could be found, so that 56 patients were available for the final analysis. Patient’s age ranged from 11 to 61 years (mean age = 34.3 ± 12.5 years). Twenty-one patients presented with a ruptured AVM, and 35 with an unruptured malformation. Preoperative embolization was used as an adjunct in seven of our patients. The hypotension and control group consisted of 28 patients each, and did not differ with respect to age, gender, body weight, height and the proportion of ruptured AVMs (Table 1). However, patients of the hypotension group presented with a significant (*p* = 0.025) lower modified Rankin scale grade (mRS) on admission (mRS = 0 [0; 1] versus 1 [0; 2]) and tended to be more often without disability (19 versus 11 patients, *p* = 0.061) than the control group patients (Table 1). In contrast, hypotension group patients showed significantly higher AVM size grading (15 versus 6 patients with AVM size of 3–6 cm, *p* = 0.027) and Spetzler-Martin grading (grade 3 [2; 3] versus grade 2 [1.25; 2], *p* <  0.001) than control patients (Table 1). A high Spetzler-Martin grade as high as 3 or 4 occurred significantly (*p* = 0.001) more often in the hypotension (18 patients) than in the control group (5 patients).

**Table 1:** Preoperative data of the study cohort

	Hypotension group	Control group	***p***-value
**Epidemiology**			
Number of patients	28	28	
Gender ratio (female: male)	14:14	14:14	
Age (years)	35 ± 12	34 ± 13	
Height (cm)	173 ± 9	172 ± 10	
Weight (kg)	74 ± 19	68 ± 17	
**Characteristics of the AVM**			
Clinical presentation:			
pts. with ruptured AVM	7	14	0.098
mRS on admission	0 [0; 1]	1 [0; 2]	0.025
pts. without disability (mRS = 0)	19	11	0.061
AVM size:			
< 3 cm	13	22	0.027
3–6 cm	15	6	
Venous drainage of the AVM:			
Superficial	20	22	0.32
Deep	13	8	
AVM location:			
within eloquent area	18	13	0.35
Spetzler-Martin grading:			
grading on admission:	3 [2; 3]	2 [1.25; 2]	< 0.001
pts. with grade 1 & 2	10	23	0.001
pts. with grade 3 & 4	18	5	

Data are shown as mean ± standard deviation in case of normal distribution or as median [25%, 75% percentile] otherwise. *AVM* arteriovenous malformation, *mRS* modified Rankin Scale, *pts.* patients

### Course of arterial blood pressure

**Fig. 1:**
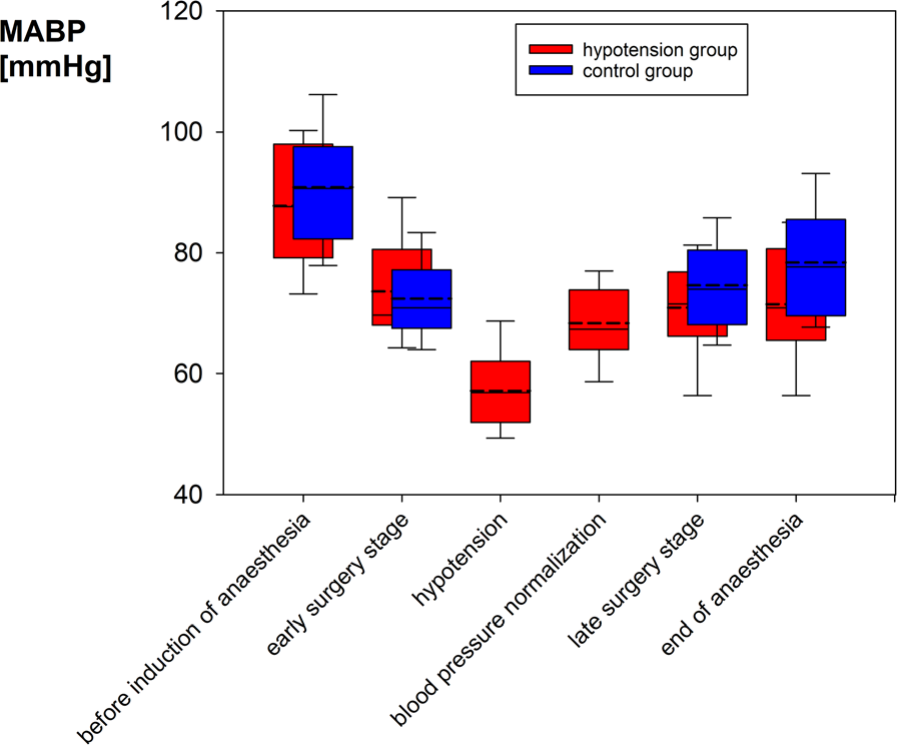
Intraoperative course of mean arterial blood pressure (MABP) in the hypotension group (*n* = 28 patients) and the control group (*n* = 28). The boxplots indicate the median, 10th, 25th, 75th and 90th percentiles, with a dashed line showing the mean value. Early and late surgery stage refer to the first and last third of the surgical procedure. Groups do not differ in their ABP, except for the end of anaesthesia. In the control group, neither hypotension nor blood pressure normalization was performed by definition

During CAH, the systolic and mean arterial blood pressure (MABP) were lowered to 82 ± 7 and 57 ± 7 mmHg, respectively, for a duration of 58 [26; 107] minutes. At the end of anaesthesia, MABP was lower in the hypotension group (71 ± 10 versus 78 ± 10 mmHg); otherwise, there was no difference in ABP between groups (Fig. 1). General anaesthesia was performed with isoflurane (*n* = 46) or propofol (*n* = 10).

We found a negative correlation between the Spetzler-Martin grading and the mean ABP (*r* = − 0.288; *r*^*2*^ = 0.083; *p* = 0.031) and a positive correlation between the size of the AVM and the duration of hypotension (*r* = 0.517; *r*^*2*^ = 0.267; *p* = 0.005).

### Methods used to induce and terminate CAH

CAH was induced most often by urapidil, either as a sole agent (*n* = 11 patients) or in combination with a deepening of general anaesthesia (*n* = 6). In another 3 patients, urapidil was combined with one or two other antihypertensives. In further 4 patients, CAH was induced by deepening of general anaesthesia without application of antihypertensive drugs. In 4 cases, no clear method could be identified.

Neuroanaesthetist and neurosurgeon jointly decided on a systolic ABP target of 80 mmHg during CAH. This target could be achieved in 35.7% of patients during the entire period of CAH and in 14.3% of patients during more than 50% of the time. In the remaining 50% of patients, the target was either never reached or in less than 50% of the time only. The mean ABP was below 60 mmHg in 18 out of the 28 patients (68%).

CAH was most frequently (*n* = 12) terminated by administration of akrinor, which is a mixture of 200 mg cafedrine and 10 mg theodrenaline. In addition, CAH was finished by using akrinor combined with noradrenaline (*n* = 2), stopping the administration of urapidil (*n* = 3) or nitroglycerine (*n* = 1), a reduction of general anaesthesia (*n* = 4) or a combination of akrinor and reduction of general anaesthesia (*n* = 1). In three cases, no clear method could be identified, and in two patients hypotension was continued beyond the end of surgery. None of the patients required catecholamines to support blood pressure at the end of surgery or during transfer to intensive care. For the postoperative period of 24 h, an upper limit for systolic blood pressure was set in 15 patients, with a target of 120, 100 and 90 mmHg in 3, 10, and 2 patients, respectively.

### Blood loss and postoperative outcome

Intraoperative blood loss was 375 [200; 775] ml (range from 50 to 3400 ml), with significantly (*p* = 0.002) more blood loss (by 300 ml, Table 2) in the hypotension group than in the control group. Blood transfusion was necessary in 2 cases in the control group and in 7 cases in the hypotension group. The lowest intraoperative haemoglobin concentration was 11.7 ± 2.1 (range from 7.5 to 15.8) g/dl, with Hb tending to be slightly lower (*p* = 0.15) in the hypotension than in the control group (11.3 ± 2.2 g/dl vs. 12.1 ± 2.0 g/dl, Table 2). The lowest peripheral oxygen saturation was 99 [98; 199] %, with no difference between the groups.

**Table 2:** Intra- and postoperative characteristics of both groups

	Hypotension group	Control group	***p***-value
**Intraoperative findings**			
Duration of surgery (h)	4.4 ± 1.3	3.3 ± 0.9	< 0.001
Duration of anaesthesia (h)	5.4 [4.7; 6.5]	4.3 [4.1; 5.2]	0.013
Blood loss (ml)	500 [300; 950]	200 [100; 400]	0.002
Lowest Hb conc. (g/dl)	11.3 ± 2.2	12.1 ± 2.0	0.15
Lowest SpO_2_ (%)	99 [99; 99]	99 [98; 100]	0.67
**Postoperative findings**			
mRS after surgery	1 [0; 2]	0 [0; 2]	0.87
Any new neurological deficits			
Transient	16	7	0.03
Permanent	8	2	0.08
Any new hemiparesis	9	2	0.04

Data are shown as mean ± standard deviation in case of normal distribution or as median [25%, 75% percentile] otherwise. *Hb conc.* haemoglobin concentration, *SpO*_*2*_ peripheral oxygen saturation, *mRS* modified Rankin Scale

**Fig. 2:**
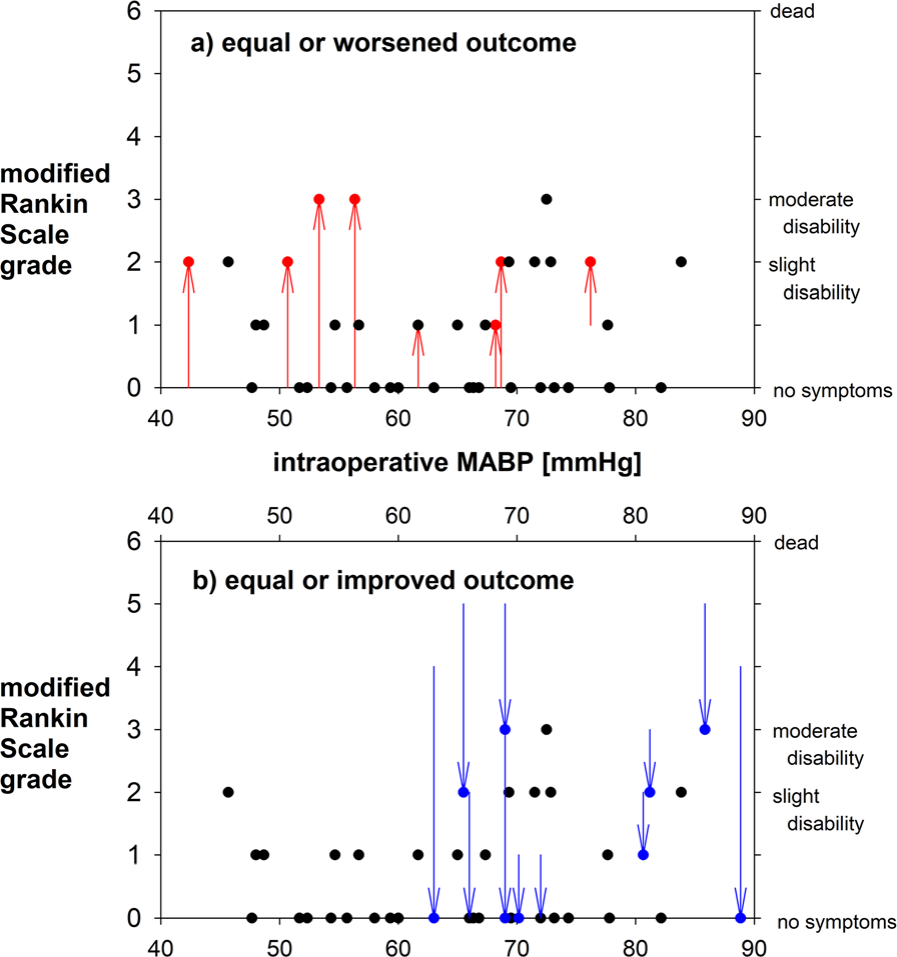
The pre- and postoperative modified Rankin Scale (mRS) grade is shown in relation to the intraoperative mean arterial blood pressure (MABP). Postoperative mRS grades are shown as circles. They are displayed in black colour in patients in which the mRS grade remained unchanged. Otherwise, arrows indicate the change from pre- to postoperative mRS grades. Patients with equal or worsened outcomes (red arrows) are shown in the upper part, and patients with equal or improved outcomes (blue arrows) in the lower part

In the hypotension group, the duration of surgery and anaesthesia was significantly longer (by 66 min) as compared to the control group (Table 2). Follow up was 8 [3; 57] months. None of the patients died (Fig. 2), developed acute renal failure or myocardial infarction. There was no difference in the overall complication rate between the groups. However, the number of patients with either transient (16 versus 7 patients) or permanent (8 versus 2 patients) neurological deficits was significantly higher in the hypotension group than in the control group, respectively (Table 2). A significant correlation between the occurrence of postoperative neurological deficits and both the mean ABP (*r* = − 0.387; *r*^*2*^ = 0.15; *p* = 0.003) and the duration of CAH (*r* = 0.322; *r*^*2*^ = 0.104; *p* = 0.016) was observed.

**Fig. 3:**
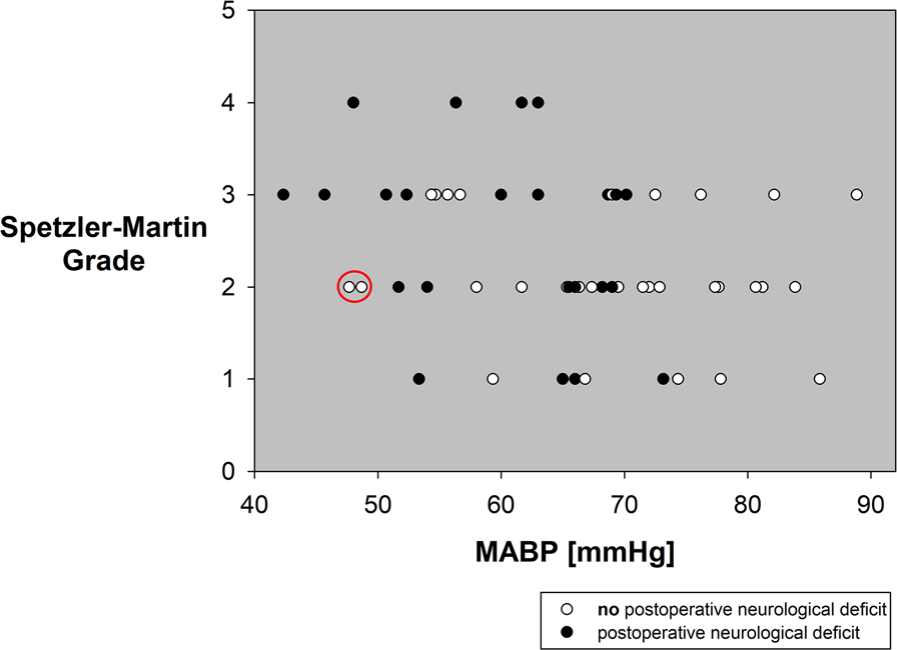
Postoperative occurrence of neurological deficits depending on the intraoperative mean arterial blood pressure (MABP) and the Spetzler-Martin grading. There were no neurological deficits when the MABP was > 75 mmHg. Patients with and without postoperative neurological deficits are shown as black or white circles, respectively. Two patients with a MABP ≤51 mmHg developed no deficit (as marked by the red circle). In both, hypotension was performed for a short duration (5 and 10 min) only

**Fig. 4:**
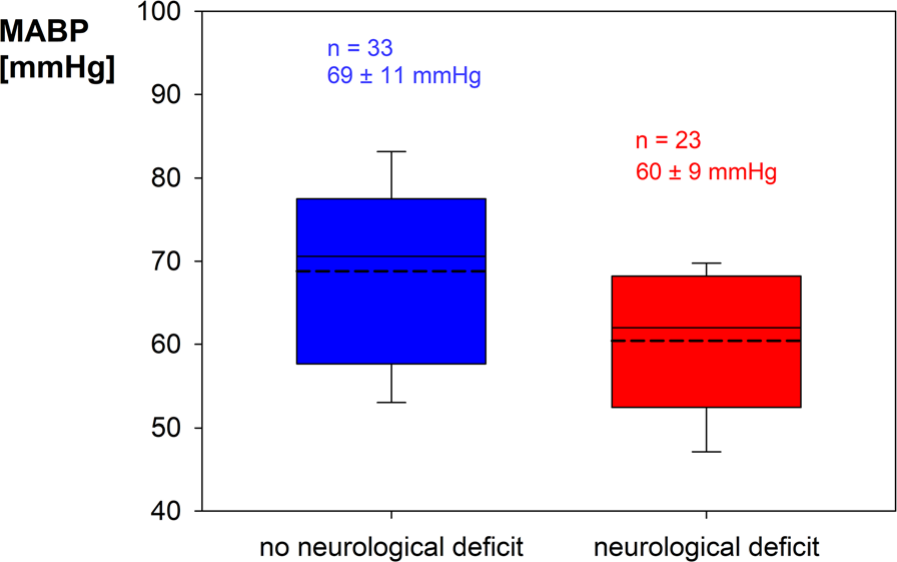
Mean arterial blood pressure (MABP) in comparison between patients with and without postoperative neurological deficits. Boxplots indicate median, 10th, 25th, 75th and 90th percentile. The mean value is shown as dashed line

**Fig. 5:**
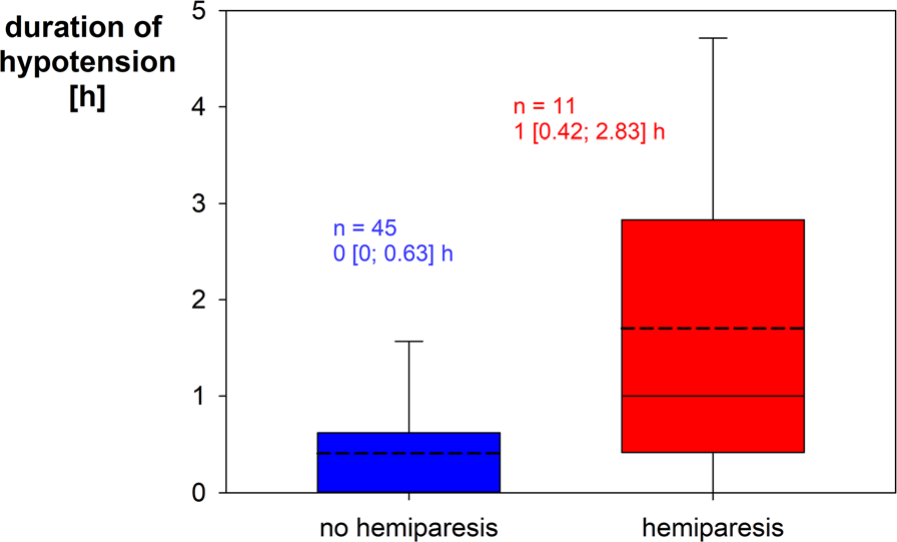
Duration of hypotension comparing patients with and without the occurrence of postoperative hemiparesis. Control group patients had a duration of hypotension of 0 h by definition. Boxplots indicate median, 10th, 25th, 75th and 90th percentile. The mean value is shown as dashed line

Comparing patients with and without postoperative neurological deficits, the former had higher values on the Spetzler-Martin scale (3 [2; 3] vs. 2 [2; 3]; *p* = 0.082; Fig. 3), a significantly lower mean ABP (60 ± 9 mmHg vs. 69 ± 11 mmHg; *p* = 0.003; Fig. 4) and spent approximately 30 min longer in CAH (*p* = 0.017). In addition, patients with postoperative hemiparesis showed a significant lower mean ABP (58 ± 9 mmHg vs. 67 ± 10 mmHg; *p* = 0.01) and CAH was approximately 1 hour longer (*p* = 0.004, Fig. 5). Even when considering the hypotension group alone, the duration of CAH was significantly (*p* = 0.017) longer in case of postoperative hemiparesis (2.1 ± 1.7 h vs. 1.0 ± 0.7 h).

### Predictors of postoperative neurological deficits

**Fig. 6:**
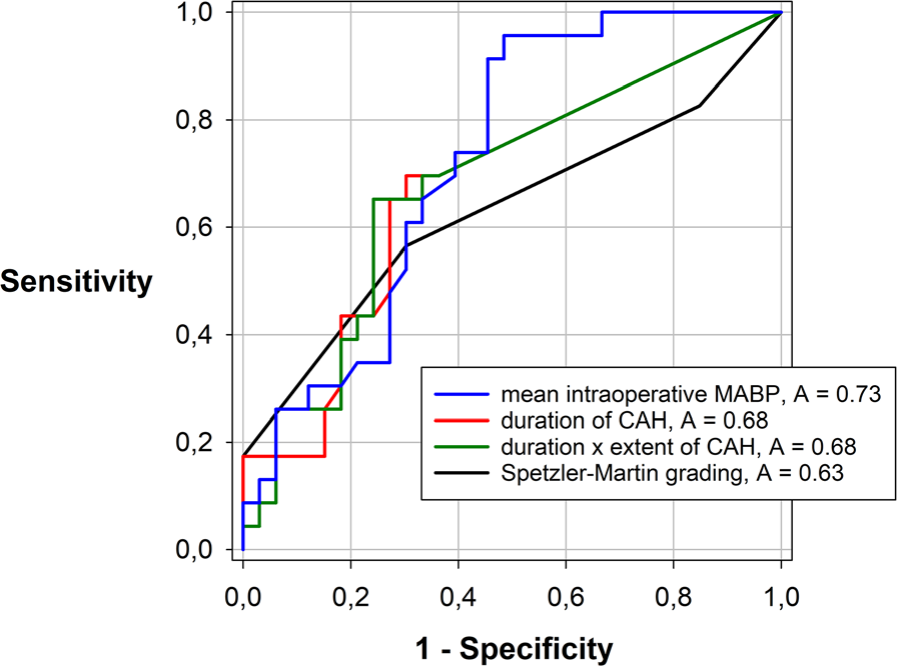
Receiver Operating Characteristic (ROC) of four parameters to predict an early neurological deterioration, i.e. any non-pre-existing neurological deficit. ROC analysis was significant for the three parameters mean intraoperative MABP (*p* < 0.001), duration of CAH (*p* = 0.03) as well as for the product of duration and extent of CAH (*p* = 0.02), but not for the Spetzler-Martin grading (*p* = 0.10). MABP = mean arterial blood pressure, CAH = controlled arterial hypotension, A = area under the curve

Best Subset Regression analysis yielded Spetzler-Martin grading, duration of CAH, intraoperative mean ABP, blood loss and lowest intraoperative haemoglobin concentration as potential predictors for the occurrence of any new postoperative neurological deficit. However, the final Backward Stepwise Regression Analysis showed that the occurrence of postoperative neurological deficits could be best predicted by intraoperative mean ABP (coefficient of determination *R* = 0.39, adjusted *R*^*2*^ = 0.13, standard error of the estimate = 0.47, *p* = 0.003, Table 3), whereas the above-mentioned variables did not significantly add to the ability to predict outcome. ROC analysis showed the highest area under the curve (AUC) to predict any new neurologic deficit for intraoperative mean ABP (AUC = 0.73, *p* <  0.001), followed by the duration of CAH (AUC = 0.68, *p* = 0.03) and the product of duration and extent of CAH (AUC = 0.68, *p* = 0.02). In contrast, the AUC for Spetzler-Martin grading (0.63) was not significant different from 0.5 (Fig. 6). An intraoperative mean ABP of < 72 mmHg predicted a postoperative neurological deficit with a sensitivity of 0.96 and a specificity of 0.45. If a mean ABP threshold of 65 mmHg was specified instead, a sensitivity of 0.57 and a specificity of 0.70 resulted.

**Table 3:** Predictors of outcome

Outcome	Backward stepwise regression			Predictor	*P*-value
(Independent variable)	R	Adjusted R^2^	SE	(Dependent variable)	
Any early, non-pre-existing, neurological deficit	0.39	0.15	0.47	Intraoperative mean ABP	0.005
Any transient or permanent hemiparesis	0.52	0.27	0.35	Duration of CAH	< 0.001
Long-term modified Rankin Scale	0.54	0.29	0.88	mRS before surgery	< 0.001
				Intraoperative mean ABP	0.03
				Lowest haemoglobin conc.	0.014

Prediction of outcome as determined by backward stepwise regression. R = correlation coefficient, adjusted R^2^ = adjusted R^2^ according to the degrees of freedom. *SE* standard error of the estimate

The occurrence of postoperative hemiparesis was best predicted by the duration of hypotension (Backward Stepwise Regression with coefficient of determination *R* = 0.52, adjusted *R*^*2*^ = 0.27, standard error of the estimate = 0.35, *p* <  0.001, Table 3), whereas other variables such as age, preoperative seizures, Spetzler-Martin grading, and mean ABP did not add significantly to the ability to predict hemiparesis. The ROC analysis showed for a period of hypotension of > 15 min a sensitivity of 0.82 and a specificity of 0.64 to predict hemiparesis with an AUC of 0.77. When a duration of 30 min was selected instead, a sensitivity of 0.64 and a specificity of 0.71 was obtained. Long term outcome in terms of the modified Rankin Scale was best predicted (Backward Stepwise Regression with coefficient of determination *R* = 0.54, adjusted *R*^*2*^ = 0.29, standard error of the estimate = 0.88, *p* <  0.001, Table 3) by the three independent variables mRS before surgery, intraoperative MABP and the lowest haemoglobin concentration (Table 3).

## Discussion

We hypothesized that CAH would lower blood loss compared to a procedure without CAH. Since it is unfeasible to operate the same case without CAH and then again with CAH, it is impossible to determine whether the use of CAH lowered an expected blood loss or not. We expected that CAH would have no negative effect on postoperative outcome but observed an association with more neurological deficits. Complications of surgery must be seen in the light of the complication rate of untreated AVMs, which carry a life-long risk for stroke due to rupture of about 2 to 4% per year [12, 18]. Therefore, surgery appears justified when considering that AVM rupture and subsequent intracerebral haemorrhage are associated with high morbidity and mortality. It is important to keep in mind that CAH was applied in a subgroup of difficult cases with a higher surgical risk in order to prevent excessive blood loss and to minimize the expected frequency of neurological deficit.

In this context, we would like to discuss two possible scenarios, one is cerebral injury caused by CAH and the other the prevention of worse outcomes by CAH.

### Cerebral injury due to CAH

The observed correlation between degree (or duration) of CAH and neurological outcome suggests a relationship between hypotension and outcome. Both depth and duration of CAH also turned out to be predictors for the incidence of neurological deficits and seemed to have more influence than the location, graduation or size of the AVM. However, these findings cannot distinguish whether there is an association or a causal relationship between hypotension and outcome. To clarify this matter, a prospective randomized-controlled study would be necessary. However, such a study would likely be considered unethical since the non-CAH group would be at an increased risk of bleeding and adverse surgical conditions.

The AVM serves as a low-pressure, high-flow shunt, so that cerebral ischaemia can develop in the surrounding tissue due to the resulting arterial hypotension and steal phenomena [24]. Therefore, the deliberate use of CAH as a technique for minimising blood loss must be used with caution and serious consideration given to the haemodynamic changes within the AVM and the adjacent tissue that occur during and early after AVM occlusion [24]. However clinical studies have shown that despite the lowered perfusion pressure, severely ischemic hypoxic areas are uncommon in the cortex adjacent to AVMs [22, 23, 42]. But in few patients, which are prone to hyperaemic complications, some areas of local low blood anoxia may exist [22, 23].

The MABP of 57 ± 7 mmHg observed during hypotension in our study is well below the lower threshold of cerebral autoregulation, which is unknown in a given patient but commonly assumed to be around 70 mmHg in the normotensive adult, although autoregulation limits are reported to be subject to interindividual variability [2, 8]. We can therefore expect a reduced CBF during the period of CAH, although the extent of flow reduction remains unknown. Since the brain is protected by luxury perfusion, cerebral ischemia is thought to occur in a supine position below a MABP of 45–55 mmHg [8], which was not reached in our study. These blood pressure limits must always be considered in the context of the reference level of measurement and the patient’s position. Following our clinic standards, we referenced the arterial line to the right cardia atrium and not the meatus acusticus externus. The latter reflects the driving forces of CPP much better, especially when the head is positioned above heart level, however that was not the case with our patients.

A leftward shift of the autoregulation curve in the brain tissue around the AVM has been reported, predisposing these patients to postoperative hyperperfusion while providing some resistance against hypoperfusion [15, 22]. Moreover, profound arterial hypotension would cause a rather global than focal cerebral ischemia with corresponding symptoms. In contrast, the neurological deficits we observed after surgery were focal rather than global. We therefore conclude that the patients treated with CAH suffered not from global cerebral ischemia and regard the connection between hypotension and outcome as an association and not as a causal relationship.

### CAH to enable resection and to prevent worse outcomes

The patient cohorts were not comparable with respect to their AVMs: Patients treated with CAH showed higher Spetzler-Martin grading mainly due to their larger AVM size. We can therefore assume that the patients in the hypotension group suffered from AVMs, which were more difficult to resect with a higher risk of neurological complications. In fact, because of the expected difficulty, a higher incidence of side effects was anticipated, with these anticipated difficulties being the main reason for wishing induced hypotension. In general, there were no neurological deficits when the MABP was > 75 mmHg regardless of the Spetzler-Martin grading.

Since all of our patients were operated by the same neurosurgeon, differences in the duration of surgery and in blood loss may be regarded as indicators for the difficulty/complexity of AVM-resection. Surgery in the CAH group lasted on average 66 min longer and was associated with higher blood loss than in the control group, suggesting a more difficult/complex resection in the hypotension group. This can be attributed to the fact that the neurosurgeon wished for the initiation of CAH because he experienced or anticipated difficulties for example due to bleeding. In the vast majority of patients, CAH was not started as a reaction to diffuse bleeding, but it was a preoperative decision in nearly all cases. Usually, the ABP range was already decided by anaesthetist and neurosurgeon prior to skin incision. Due to the large experience of the surgeon, it was usually no problem to decide which case might pose difficulties with haemostasis. In general, CAH was not a panic reaction to some surprising event but a well-planned resection policy.

AVM-vessels are fragile and difficult to coagulate [17]. According to Michael T. Lawton [20], “AVM bleeding can be so brisk that it overwhelms the neurosurgeon and spirals out of control.” In addition, he wrote that he “can think of nothing in neurosurgery more challenging than salvaging these desperate situations.” CAH is thought to reduce blood loss and to help the surgeon to keep control over the site of surgery [15]. Therefore, it is likely that the initiation of CAH prevented more blood loss in difficult cases where blood loss was already increased instead of causing blood loss as our results suggest at first glance.

AVM resection is associated with considerable morbidity and mortality, with higher Martin-Spetzler grading resulting in worse outcomes [29, 37]. No case fatalities occurred in our study, whereas other authors reported a mortality rate of 2.7% [39], as the same surgeon found in his total series [30]. However, transient and permanent neurological deficits occurred in 41 and 18% of our patients, respectively. Neurosurgical studies assume that these deficits are more likely to be caused by surgery and AVM location, based on the increasing risk depending on size and grading [30, 37, 39]. In addition, anaesthesiologic studies report that even low blood pressure values are tolerable over a certain period. However, there is no agreement on the actual blood pressure values and duration [5, 19, 33, 40, 41, 43]. Due to their heterogeneity, these studies are only partially comparable with this study and with each other. It is clear, however, that the individual limits of (possibly impaired) cerebral autoregulation for the individual patient remain unknown [33]. Even though the autoregulatory curve is shifted to the left in the brain areas adjacent to the AVM [22], it can be assumed that in this study the lower limit of the autoregulation was undercut several times and therefore a triggering or worsening of neurological deficits is possible [10]. However, we suspect that these complications are rather due to AVM location and surgery but not due to arterial hypotension, since the neurological symptoms were rather focal than global.

### Implementation of controlled arterial hypotension

The decision for or against CAH was made jointly by neuroanaesthetist and neurosurgeon based on the shared responsibility for the patient. Certainly, blood pressure management is the responsibility of the anaesthetist and in many countries, it is therefore unusual for the surgeon to be involved in blood pressure management. Especially in the situation of threatened or actual bleeding during resection of AV malformations, we believe that the team decision has proven its worth at our hospital.

No method to induce or terminate controlled arterial hypotension proved superior compared to the other methods used in this study. Although CAH is a routine task performed by anaesthesiologists, its implementation is by no means trivial. MABP was lowered to some extent in all patients of the hypotension group, but the target MABP was never reached in 18% of patients. Despite the administration of highly effective vasoactive drugs, precise control of blood pressure is often difficult, especially if a too pronounced reduction in blood pressure is to be avoided. No consensus exists in the literature as to which agents should be used [6], but from a pathophysiological point of view, drugs that lead to cerebral vasodilation should be avoided as they can exacerbate existing vascular steal effects and increase intracranial pressure [24]. Examples are nitroglycerine, nitroprusside and volatile anaesthetics at high concentrations (> 1.5 MAC). Preferred drugs to induce and maintain CAH are remifentanil, urapidil, esmolol, labetolol, or nicardipine [24]. Often the effect of non-short acting drugs such as urapidil must be reversed by catecholamines at the end of the CAH phase. The patient population of our study is too small to investigate whether certain drugs are better suited for CAH than others.

No consensus exists as to which target MABP should be achieved during CAH [8, 41]. Hernesniemi proposed a systolic ABP of usually 100 mmHg, which is lowered to 60–70 mmHg for a short interval in some AVM cases [17], as part as of his so-called “dirty coagulation” technique for the event of difficult haemostasis. Otherwise, we did not find any specific recommendations about the extent of CAH for AVM resection in the literature.

Studies on perioperative myocardial infarction have brought the intraoperative course of blood pressure more into the focus of anaesthesiologists. Accordingly, intraoperative MABP below 55–70 mmHg is associated with myocardial injury, myocardial infarction, renal injury, and death [13, 31, 40]. Our AVM patient population, with a mean age of 34 years, was approximately 20–30 years younger than the patients included in the above-mentioned studies on the relationship between intraoperative hypotension and postoperative mortality or morbidity [13, 31, 40]. Therefore, our patients presumably suffered less frequently from cardiovascular diseases typical of older age and could explain why in our study, neither renal failure nor myocardial infarction occurred. However, we cannot exclude myocardial damage in asymptomatic patients because troponin determination was only performed in case of cardiac symptoms at that time and > 65% of perioperative myocardial infarctions have been reported to occur without symptoms [7]. A relationship between intraoperative hypotension and postoperative stroke has been described by some authors [1, 8, 9, 21, 38], but could not be confirmed by others [16]. Based on the available literature, the Perioperative Quality Initiative established a consensus statement [31] that “intraoperative MABPs below 60-70 mmHg are associated with myocardial injury, acute kidney injury, and death.” In addition, they conclude that “there is increasing evidence that even brief durations of systolic arterial pressure < 100 mmHg and MABP <60-70 mmHg are harmful during non-cardiac surgery.”

Since cerebral oxygen supply is determined by cerebral blood flow, haemoglobin concentration and blood oxygen saturation, both anaemia and hypoxaemia could contribute to cerebral hypoxia. No hypoxia but some “mild” degree of anaemia (11.7 ± 2.1 g/dl) occurred in our patients despite transfusion of packed red blood cells. We believe that we have performed blood gas analyses often enough to detect the nadir of Hb concentration and identified the lowest intraoperative Hb concentration as an independent predictor of long-term outcome.

### Limitations of this study

A major strength of our study is that surgery was performed by a single surgeon only, thus eliminating intersubject (perhaps better inter-surgeon) variability in outcome. However, it is limited by the retrospective design and sample size. The archived anaesthesia protocols only go back to the year 2003, so only 56 of the 226 patients operated on in Bonn since 1990 could be included. Hence, results should be interpreted with caution and should not easily be generalized.

The blood pressure in the postoperative course can also influence the treatment outcome. For example, postoperative increases in blood pressure can lead to neurological deterioration with cerebral oedema, based on the Normal Perfusion Pressure Breakthrough Theory [36]. This has been investigated and addressed in various publications [15, 22, 24, 27], but was not the aim of our study. Instead, we specifically focussed on the intraoperative course of ABP.

Age is a predictor for outcome in AVM [37]. Since age was similar in the hypotension and control group, both groups were well comparable. However, some differences in the AVM-characteristics (see Table 1) might contribute to bias and are a limitation of the study.

Current management options for AVMs include microsurgical resection, embolization, stereotactic radiosurgery, and medical treatment (i.e. observation with pharmacological therapy for neurological symptoms as needed) [4], whereby no definitive treatment guidelines exist [34]. While intervention is recommended in ruptured AVMs with complete nidal obliteration as the therapeutic goal to strive for [4], medical management alone has been reported to be superior to a combined management including intervention in ARUBA, a multicentre, randomised controlled trial of unruptured AVMs [25, 26]. This has led to controversial discussions about the different treatment options for unruptured AVMs, details of which can be found elsewhere [4, 11]. Our results refer to microsurgical resection with or without preceding embolization and cannot be transferred to other therapeutic options such as stereotactic radiosurgery or embolization alone.

## Conclusions

In case of expected difficulties during AVM-resection, a team decision to apply CAH can be made. However, the outcomes in our patients operated with CAH are worse than in those operated without CAH. It remains unclear, whether this reflects (only) the complexity of the case, or is (also) an effect of the CAH. Therefore, CAH should only be applied with strict indications and as mild and short as possible.

## Data Availability

The datasets used and/or analysed during the current study are available from the corresponding author on reasonable request.

## Abbreviations

ABP: Arterial blood pressure
AUC: Area under the curve
AVM: Arteriovenous malformation
CAH: Controlled arterial hypotension
CBF: Cerebral blood flow
Hb: Haemoglobin
SpO_2_: Peripheral oxygen saturation
MABP: Mean arterial blood pressure
MAC: Minimal alveolar concentration
mRS: modified Rankin scale
ROC: Receiver operating characteristic
